# Analysing RNA-kinetics based on folding space abstraction

**DOI:** 10.1186/1471-2105-15-60

**Published:** 2014-02-28

**Authors:** Jiabin Huang, Björn Voß

**Affiliations:** 1 Genetics & Experimental Bioinformatics, Faculty of Biology, University of Freiburg, Schänzlestr. 1, 79104, Freiburg, Germany

**Keywords:** RNA, Folding space, Kinetics, Abstraction

## Abstract

**Background:**

RNA molecules, especially non-coding RNAs, play vital roles in the cell and their biological functions are mostly determined by structural properties. Often, these properties are related to dynamic changes in the structure, as in the case of riboswitches, and thus the analysis of RNA folding kinetics is crucial for their study. Exact approaches to kinetic folding are computationally expensive and, thus, limited to short sequences. In a previous study, we introduced a position-specific abstraction based on helices which we termed helix index shapes (*hishapes*) and a *hishape*-based algorithm for near-optimal folding pathway computation, called HiPath. The combination of these approaches provides an abstract view of the folding space that offers information about the global features.

**Results:**

In this paper we present HiKinetics, an algorithm that can predict RNA folding kinetics for sequences up to several hundred nucleotides long. This algorithm is based on RNAHeliCes, which decomposes the folding space into abstract classes, namely *hishapes*, and an improved version of HiPath, namely HiPath2, which estimates plausible folding pathways that connect these classes. Furthermore, we analyse the relationship of *hishapes* to locally optimal structures, the results of which strengthen the use of the *hishape* abstraction for studying folding kinetics. Finally, we show the application of HiKinetics to the folding kinetics of two well-studied RNAs.

**Conclusions:**

HiKinetics can calculate kinetic folding based on a novel *hishape* decomposition. HiKinetics, together with HiPath2 and RNAHeliCes, is available for download at http://www.cyanolab.de/software/RNAHeliCes.htm.

## Background

RNA molecules play vital roles in the cell, and their function is often determined by structural properties. These properties may be static, such as structural motifs, or dynamic, such as the ability to adopt different conformations as riboswitches do. The latter emphasises the importance of studying RNA folding kinetics, which is the dynamic behaviour of RNA structure over time.

Most approaches to the stochastic simulation of RNA folding kinetics can be described as Monte Carlo simulations [1-3] or continuous time Markov chains (CTMC) [4,5]. A Monte Carlo simulation requires a large number of samples of individual trajectories to achieve accuracy, rendering these methods computationally expensive. The same holds true for CTMC-based simulation, as long as it is based on a complete enumeration of the folding space. The program TREEKIN[4] implements a CTMC-based simulation, and for short sequences (e.g., up to 30 nt), can simulate exact folding kinetics. For longer sequences, however, the exponential growth of the underlying state space requires restricting the analysis to a subset of the folding space. For this purpose so called “macrostates” were introduced in [4], each of which can be seen as a local minimum and all structures that are connected to it by a gradient walk. A macrostate is represented by its local minimum secondary structure. The problem that arises from the macrostate definition is that neighbouring macrostates cannot easily be identified. The program TREEKIN uses BARRIERS to compute saddle points connecting macrostates and the corresponding transition rates. The dependence on BARRIERS limits this approach to sequences of moderate length (up to 100 nt), which can be partially overcome by restricting the analysis to conformations within a specified energy range above the minimum free energy. To overcome the length restriction and reduce the computational burden Tang et al. [6] use a sampling strategy called probabilistic Boltzmann-filtered suboptimal sampling. In their approach, sampled structures are connected by transition paths computed using a simple greedy algorithm [7]. These transition paths are weighted with their barrier energy. The procedure may be suboptimal in two ways: first, the sampling may miss important structures in the folding space, and second, the greedy pathway prediction may overestimate energy barriers and lead to inaccurate transition rates.

The computation of exact, globally optimal folding pathways between any two secondary structures (e.g., BARRIERS[1,8]) is NP-hard [9]. Many heuristic approaches for computing folding pathways have been proposed. The first approach was proposed by Morgan and Higgs [10] by selecting the least “clashing” base-pairs as the next intermediate structure from a set of neighbouring structures. Subsequently, the idea was extended by Flamm *et al.*[11]. Instead of selecting the best structure as the next intermediate structure, the *k* best candidates are maintained during the folding pathway construction (breadth first search, BFS). In contrast to these direct path heuristics (intermediate structures contain only base pairs that are also present in the start or target structure), Dotu *et al.*[12] presented a heuristic including indirect paths. Li *et al.*[13] proposed an evolutionary algorithm in which a pathway is represented by an action chain that is mutated by different strategies to find a better solution.

In general there are two central challenges in CTMC-based folding simulations for RNA. How can the energy landscape be decomposed in a complete, compact and non-heuristic way? And how can the transition rates between partitions be calculated accurately and efficiently?

Our contributions in this paper address these challenges. In previous work [14], we introduced *hishapes* as classes of structures sharing the same helices. These *hishapes* intrinsically decompose the folding space into disjoint classes, which are represented by the member with minimum free energy, called the *hishrep*. This partitioning is complete and non-heuristic, and its coarse-graining can be adjusted based on its abstraction levels, which differ in the type of structural elements they consider. Here, we analyse the degree to which *hishapes* overlap with locally optimal structures. Additionally, we provide a new folding space restriction, called strictly negative structures, that eliminates suboptimal structures with positive energy substructures. We present HIPATH2 as an improved version of HIPATH[14] and show that it computes lower energy barrier folding pathways for most cases in our benchmark set. Finally, we combine these methods in HIKINETICS, a tool for simulating RNA folding kinetics using strictly negative *hishapes* for the folding space decomposition and energy barriers estimated by HIPATH2 to derive transition rates using Arrhenius’ equation. We apply our novel kinetic analysis tool termed HIKINETICS to two well-studied RNAs.

## Results and discussion

### *Hishapes* revisited

**Figure 1:**
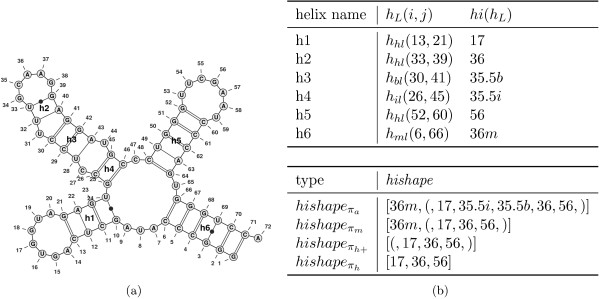
**Helices, *helix indices *and *hishapes *(a) example secondary structure, (b) properties of its helices and the resulting *hishapes *. ***hl*, *bl*, *il*, and *ml* refer to hairpin, bulge, internal and multiple loop, respectively. The letters *m*, *b*, and *i* appended to *helix indices* within *hishapes* indicate the loop type (multiple, bulge, and internal loop, respectively). *Helix indices* without suffixes represent hairpin loops. Pairs of brackets in a *hishape* provide nesting information within multiloops. Picture taken from [14].

We begin with a brief recapitulation of the central concepts and notations of *hishapes*. For formal definitions, we refer the reader to our previous manuscript [14]. For *hishapes*, we consider an RNA secondary structure as a set of helices terminated by loops (internal, bulge, multiple and hairpin loops). The innermost base pair (*i*,*j*) of a helix corresponds to the closing base pair of the terminating loop, and we define (*j*-*i*)/2 to be the *helix index* of this helix. Additionally, we mark the *helix index* with *m*, *b*, or *i* for multiple, bulge, or internal loop, respectively. Using a mapping function *π*, we can now map any secondary structure to a *helix index* shape (*hishape*), which is simply a list of *helix indices*. Figure 1 illustrates the relationship among helices, *helix indices* and *hishapes*. To provide different levels of abstraction, we make use of different mapping functions. The function *π*_*h*_ retains only hairpin loop helices and *π*_*h*+_ additionally keeps track of the nesting within multiloops. The functions *π*_*m*_ and *π*_*a*_ extend *π*_*h*+_ through retaining multiloops and all helices, respectively. A *hishape* defines a class of similar structures, and we use the member with minimum free energy as the *hishape* representative (*hishrep*).

### Reducing the search space to strictly negative structures

The number of feasible secondary structures grows exponentially with the length of the RNA. We recently presented *hishapes*, which abstract from helix lengths and, depending on the abstraction type, also from certain loop types. Compared to suboptimal structures, the number of possible *hishapes* is dramatically reduced, but it still grows exponentially with sequence length.

**Figure 2:**
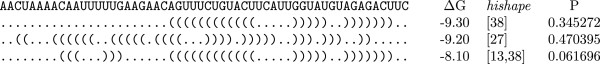
**Three best *hishapes *of the spliced leader RNA from *L. collosoma *.** The leftmost column lists *hishreps*. *ΔG* is the free energy in *kcal*/*mol* and *hishape* represents the *π*_*m*_*hishape*. *P* is the *hishape* probability. This figure was generated using 'RNAHeliCes -f examples/spliced_leader.seq -q’.

*Hishapes* provide deep insight into the folding space of an RNA molecule while keeping the output at a manageable size. Analysing one of our favourite toy examples, the Spliced Leader RNA from *Leptomonas collosoma*, we recognised that there are pairs of *hishapes* where the *hishrep* with an additional helix has a higher energy, as shown in Figure 2. Here, due to the additional helix with *helix index* 13, the energy of *hishape*[13,38] is worse than the energy of *hishape*[38].

The formation of this helix imposes an energy cost of 1.2 kcal/mol and, thus, is thermodynamically unfavourable. To eliminate such unfavourable structures, we cannot simply exclude all positive energy substructures within our recursive DP calculation. Doing so would for example disallow nearly all hairpin loops and thereby the computation of many biologically significant structures. We take the view that closed substructures within the external loop or within a multiloop must not have positive energy. We are aware that disallowing positive energy substructures within multiloops may even remove the minimum free energy (MFE) structure from the structure space. In fact, a test on 10,000 randomly selected sequences from Rfam showed that for 1.67% of the sequences, the MFE structure is removed. For these 167 sequences, the strictly negative optimal structure has a *ΔG* that is on average 0.49 kcal/mol (*σ*=0.367, *max*=2.3 kcal/mol) worse than the MFE. However, these differences are on the same scale as (or even below) the uncertainties present in the thermodynamic parameters used for computation.

A further reason we think that removing substructures with positive energy is reasonable is that they seem kinetically unfavourable. A helix nucleates by formation of the terminal hairpin loop, which is the time dominating step, and is subsequently stabilised by the stacking of base pairs. For positive energy substructures, the *ΔG* of the hairpin loop is very large, which results in a low probability of nucleation, and/or the *ΔG* of the stacking pairs is small, which renders the melting of such helices very likely. For these reasons, we believe that disallowing positive energy substructures is a reasonable method to reduce the search space, although it is a heuristic filtering.

**Figure 3:**
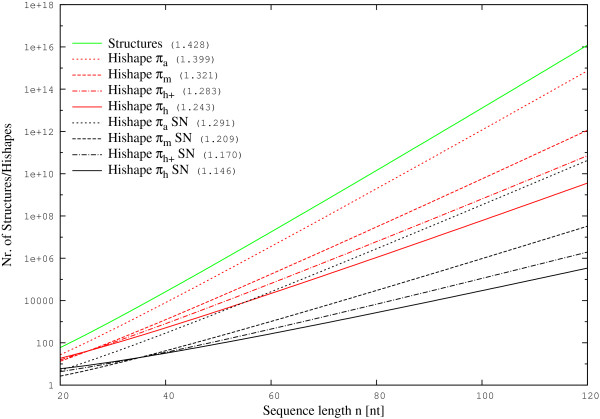
**Comparison of structure/*hishape *spaces.** All possible structures and *hishapes* were predicted for random sequences of lengths ranging from 20-120 nt, using RNASUBOPT -noLP and RNAHELICES with different abstraction levels and restricting to strictly negative (SN) structures, respectively. The average numbers of structures/*hishapes* for each length were fitted to the formula *a*×*b*^*n*^×*n*^-3/2^[15]. The numbers in parentheses give the values for *b*, which is the dominating factor in this term.

Because we can check for substructures with positive energy during the recursive calculation, this filter has nearly no computational burden. On the contrary, the reduced number of intermediate results actually speeds up the calculation. Restricting the analysis to strictly negative (SN) *hishapes* significantly reduces the search space (see Figure 3). It still grows exponentially with sequence length, but much more slowly, which is reflected by the much smaller base in the exponential growth asymptotics.

### *Hishreps* versus local optimal structures

We were interested in the question of to what extent *hishreps* overlap with the set of locally optimal structures. As described, e.g., in [16], a locally optimal structure has the lowest free energy compared with its neighbouring structures, which are the structures that differ from it by a single base pair. Because our approach disregards any structure that contains isolated base pairs, we slightly modify the concept of the neighbourhood. Commonly, a neighbour (*A*^′^) of the observed structure (*A*) is defined by adding (or deleting) a base pair in *A*. This definition also holds true for our purposes, as long as *A*^′^ does not carry a lonely base pair. If *A*^′^ does contain a single lonely base pair as the result of previously removing a base pair, then we also delete the isolated one, resulting in the structure (*A*^′′^), which will still be treated as a neighbour of *A*. Vice versa, if *A*^′^ carries an isolated base pair due to its addition we close, if possible, an adjacent base pair. The resulting structure *A*^′′^ is then a neighbour to *A*. Note that in the two described cases, *A* and *A*^′′^ differ by two adjacent base pairs. This version of the neighbourhood should be essentially the same as the 'noLP’ move set from BARRIERS.

Based on this definition, we check whether our predicted *hishreps* are locally optimal or not. Table 1 shows, for the different abstraction levels and for strictly negative *hishapes* and all *hishapes*, the fractions of *hishreps* that are local optima. Overall, the fractions are quite high, sometimes reaching 100%. The sequence for the S-box leader constitutes a negative outlier, especially in the case of strictly negative structures, where at most only 15% of the *π*_*h*_*hishreps* are locally optimal. Strikingly, strictly negative *hishreps* less frequently correspond to local minima compared to the unrestricted case. This result is somewhat counterintuitive but may be explained as follows. Filtering for strictly negative *hishapes* removes many *hishapes*. Because most *hishapes* are actually local minima, as can be seen for the unfiltered version, these *hishapes* are also affected the most strongly. Thus, the fraction of non-local optima increases in the case of strictly negative *hishapes*. So what are these non-locally optimal *hishreps*? In our opinion, they are mainly the result of replacing helices by single stranded regions. Because the formation of the removed helix would result in a neighbouring structure with better energy, the *hishrep* of the resulting *hishape* is not a local minimum.

**Table 1:** **Fractions of locally optimal *hishreps***

**Instance**	**Length**	***π***_***a***_	***π***_***m***_	***π***_***h*** **+**_	***π***_***h***_	$\pi_{a}^{\text{SN}}$	$\pi_{m}^{\text{SN}}$	$\pi_{h+}^{\text{SN}}$	$\pi_{h}^{\text{SN}}$
		**[%]**							
SL	56 nt	79.00	98.00	98.00	99.00	73.00	85.00	94.29	96.88
		90.80	24.32	21.17	18.37	56.59	2.63	2.56	2.40
attenuator	73 nt	81.00	100.00	100.00	100.00	77.00	99.00	96.00	92.00
		94.19	32.15	23.98	18.90	76.24	14.41	2.38	0.95
ms2	73 nt	84.00	98.00	98.00	97.00	82.00	89.00	80.00	81.00
		91.30	15.96	11.84	10.85	79.61	1.31	0.38	0.29
s15	74 nt	87.00	100.00	100.00	100.00	82.00	96.00	97.00	100.00
		90.63	16.45	13.30	10.96	73.21	4.23	1.28	0.85
dsrA	85 nt	77.00	98.00	98.00	99.00	71.00	97.00	98.00	100.00
		83.70	27.30	22.58	16.39	57.26	4.56	2.00	0.76
rb2	113 nt	76.00	92.00	92.00	93.00	75.00	88.00	88.00	85.00
		79.17	28.13	27.38	22.79	74.26	12.92	11.50	7.10
alpha operon	130 nt	99.00	98.00	96.00	96.00	98.00	100.00	99.00	100.00
		96.12	9.82	4.15	2.22	72.59	1.26	0.45	0.28
rb3	141 nt	76.00	99.00	99.00	99.00	76.00	99.00	99.00	98.00
		96.20	30.56	21.48	17.52	96.20	23.24	10.61	8.11
amv	145 nt	77.00	89.00	89.00	83.00	78.00	89.00	89.00	81.00
		82.80	38.03	38.03	4.56	83.87	38.03	38.03	4.45
rb4	146 nt	96.00	100.00	100.00	100.00	97.00	100.00	99.00	81.00
		89.72	10.80	8.04	5.03	20.51	2.35	1.36	0.88
rb1	148 nt	86.00	100.00	100.00	100.00	81.00	100.00	99.00	98.00
		72.27	8.87	6.61	5.67	61.36	4.84	1.66	1.21
HDV	153 nt	96.00	100.00	100.00	100.00	96.00	100.00	99.00	98.00
		87.27	37.04	5.62	2.64	87.27	26.74	2.61	1.00
thiM leader	165 nt	85.00	100.00	100.00	100.00	82.00	100.00	100.00	100.00
		98.84	24.33	15.72	9.00	86.32	18.38	7.94	5.01
rb5	201 nt	100.00	100.00	99.00	99.00	100.00	100.00	99.00	97.00
		93.46	29.07	4.06	2.24	93.46	25.06	2.24	0.78
sbox leader	247 nt	77.00	76.00	68.00	60.00	68.00	65.00	42.00	15.00
		91.67	28.36	15.70	6.76	58.62	14.84	3.11	0.42
HIV-1 leader	280 nt	39.00	71.00	58.00	56.00	38.00	52.00	38.00	38.00
		48.75	2.09	1.34	1.21	47.50	1.20	0.60	0.54
ribD leader	304 nt	91.00	100.00	85.00	85.00	88.00	92.00	76.00	70.00
		81.25	37.17	8.83	5.53	78.57	29.21	6.23	3.18
hok	396 nt	58.00	79.00	69.00	62.00	57.00	83.00	72.00	65.00
		53.70	1.42	0.70	0.51	52.78	1.38	0.60	0.42

In each cell, the upper number represents the fraction of the set of *hishreps* that are also locally optimal $\left(\frac{|ℋ\cap ℒ|}{|ℋ|}\right)$ and the lower number represents the fraction of the set of local optima that are also *hishreps*$\left(\frac{|ℋ\cap ℒ|}{|ℒ|}\right)$. We restricted the computation of *hishapes* to the best 100 and the computation of the local optima to the corresponding energy range max{ΔG(x):x∈ℋ} above the MFE. The dataset is taken from [12]. ^*SN*^ strictly negative *hishapes*.

This reasoning together with the fact that in abstraction type *π*_*a*_ the largest number of helices is taken into account, also explains to a large degree why *hishreps* for abstraction type *π*_*a*_ are less often locally optimal than *hishreps* of types *π*_*m*_, *π*_*h*+_ and *π*_*h*_.

The opposite question, “do all locally optimal structures belong to distinct *hishapes*” is easier to address. For abstractions *π*_*m*_, *π*_*h*+_ and *π*_*h*_ the structures do not have to belong to distinct *hishapes* as two locally optimal structures differing, e.g., by an internal loop, will be mapped to the same *hishape*. The situation is different for *π*_*a*_*hishapes*, as they account for differences in all loop types. Starting from any locally optimal structure, the extension and shortening of helices cannot lead to another locally optimal structure. Reaching another locally optimal structure is only possible by adding or removing complete helices or by helix interruption, i.e., the introduction of internal or bulge loops. All these events will introduce new helices into the *π*_*a*_ abstraction, thus resulting in different *hishapes*. This point is nicely reflected by the fractions of locally optimal structures that are also *hishreps* ($\frac{\left|ℋ\cap ℒ\right|}{\left|ℒ\right|}$, Table 1). While locally optimal structures have a fairly high overlap with *hishreps* of the least abstract types *π*_*a*_ and $\pi_{a}^{\text{SN}}$, the overlap drops significantly for the other abstraction types, as many local optima differ in the composition of their internal and bulge loops and are thus not retained on these abstraction levels, as described above.

### Improved barrier energy estimation

Pathways connecting alternative structures are important features of the folding space, especially when studying folding kinetics. Here, transition rates computed based on the energy barriers, which are derived from the pathways between structures, are commonly used. It has been shown that the problem of computing the globally optimal folding pathway between two structures is NP-hard [9]. In our recent publication [14], we provided an overview of current pathway estimation tools and introduced HIPATH, outperforming the other analysed methods. Here, we present an improved version, which we term HIPATH2. One of the essential features of HIPATH is that it uses a set of related *hishapes* as anchors for estimating a (near-) optimal pathway between two structures. These related *hishapes* correspond to *hishapes* that consist of individual helix indices from the start and target structures or combinations thereof. By detailed inspection of the optimal folding pathways obtained by BARRIERS, we observed that pathway intermediates sometimes carry helices with helix indices that are not identical, but very similar to the helix indices of the start or target *hishape*, differing by only a few positions. Therefore, we implemented fuzzy related *hishapes* that also take into account the neighbourhoods (in terms of the *helix index* distance) of related *hishapes*.

HIPATH2, which is based on fuzzy related *hishapes* was benchmarked against existing methods (BARRIERS[1,8], BFS[11], RNATABUPATH[12], RNAEAPATH[13] and HIPATH[14]) on 18 conformational switches taken from [12] (see Table 2). They consist of two parts: five of them are riboswitches (rb1, rb2, rb3, rb4 and rb5) taken from [17,18], and the remaining 13 are taken from PARNASS[19]. All of the algorithms were used with the same energy rules (Turner99) [20,21]. We use the “microstate” grammar [22], which corresponds to the “-d1” option of RNAEVAL from the Vienna RNA package [23]. All other parameters were kept as the defaults.

**Table 2:** **Comparison of ****BARRIERS**** (BAR), ****BFS****, ****RNATABUPATH**** (****TABU****), ****RNAEAPATH**** (EA) and ****HIPATH**

**Instance**	**Length**	**BAR**	**BFS**	**TABU**	**EA**	**HIPATH**	**HIPATH2**
			**(k = 10)**	**(n = 1000)**			
rb1	148 nt	-	24.04	24.04	23.2	**20.94**	**20.94**
rb2	113 nt	*	8.2	7.25	**6.5**	6.6	6.6
rb3	141 nt	-	22.4	17.9	17.5	**16.7**	**16.7**
rb4	146 nt	-	**16.9**	**16.9**	**16.9**	**16.9**	**16.9**
rb5	201 nt	-	24.54	24.54	**21.44**	**21.44**	**21.44**
hok	396 nt	-	28.5	29.66	**20.7**	21.1	21.1
SL	56 nt	** 11.8**	13	12.9	13	12.4	12.4
attenuator	73 nt	** 8.3**	8.7	8.6	8.7	8.6	8.5
s15	74 nt	** 6.6**	7.1	**6.6**	7.1	7.1	**6.6**
sbox leader	247 nt	*	**5.2**	**5.2**	**5.2**	**5.2**	**5.2**
thiM leader	165 nt	-	16.13	14.84	**12.3**	14	12.4
ms2	73 nt	*	**6.6**	**6.6**	**6.6**	**6.6**	**6.6**
HDV	153 nt	-	17.4	17	16.8	**15.6**	**15.6**
dsrA	85 nt	** 8**	8.3	8.2	**8**	8.3	**8**
ribD leader	304 nt	-	10.71	**9.5**	**9.5**	10.71	**9.5**
amv	145 nt	*	5.8	5.8	5.74	5.8	**5.5**
alpha operon	130 nt	*	6.5	6.5	6.1	6.5	**5.96**
HIV-1 leader	280 nt	-	9.3	11.3	**8.9**	9.3	9.3

The dataset was taken from [12,19], the results for BFS and RNATABUPATH from [12] and the results for EA from [13]. Energy barriers are given in *kcal/mol*. The *maxkeep* value *k* was 10 for BFS itself and for the BFS used within HIPATH and HIPATH2. HIPATH2 was used with auto-adjusted fuzzy related *hishape* numbers, *π*_*a*_ and *θ*=1.5. HIPATH was used with the default parameters. Bold numbers represent the minimum value for the respective sequence. The symbol "*" means BARRIERS could not be applied because either the start or the target structure was not locally optimal. The symbol "-" means computation did not finish within one day. The energy range used with RNASUBOPT for BARRIERS was determined using HIPATH2 and set to the barrier energy of HIPATH2 + 1 *kcal/mol*. Note that the results may be different from the ones shown in [14] since the used start and target structures may differ. Here we used the ones provided in [12], while in [14] we derived them for ourselves.

The results in Table 2 show that in most cases, HIPATH2, together with other methods, produces the lowest energy barrier estimates. In the four cases where exact pathways are known, the sum of errors is reduced from 1.7 to 0.8 compared to HIPATH. Compared to the second best method, RNAEAPATH, HIPATH2 produces slightly (0.1 to 0.4 kcal/mol) less optimal pathways in four cases (rb2, hok, thiM leader, HIV-1 leader). However, in eight cases it performs better by 0.14 to 2.26 kcal/mol. A major difference is found in the runtimes of the two. Table 3 compares the runtimes of HIPATH2 and RNAEAPATH. While RNAEAPATH spends approximately 837 min., HIPATH2 only needs approximately 192 min., thus being 4.4 times faster.

**Table 3:** **Runtime comparison of ****RNAEAPATH**** and ****HIPATH2**

**Instance**	**RNAEAPATH**	**HIPATH2**
rb1	38m 49s	8m 59s
rb2	10m 31s	5m 20s
rb3	25m 45s	11m 22s
rb4	0m 02s	5m 33s
rb5	14m 31s	14m 20s
hok	443m 51s	45m 13s
SL	12m 49s	1m 31s
attenuator	15m 56s	1m 15s
s15	11m 42s	1m 06s
sbox leader	24m 47s	19m 21s
thiM leader	48m 28s	7m 41s
ms2	15m 03s	0m 25s
HDV	30m 57s	9m 50s
dsrA	14m 51s	2m 10s
ribD leader	59m 33s	24m 20s
amv	15m 05s	10m 35s
alpha operon	16m 50s	4m 59s
HIV-1 leader	37m 54s	18m 01s
Total	837m 33s	192m 09s

Run times were measured as described before [14], and both programs were used with default parameters. Sequences were taken from [12,19], and all tests were run on an 8x AMD Opteron 8378 machine with 128 GB RAM under openSUSE 11.2 (x86_64).

### Simulating folding kinetics

Our approach for simulating folding kinetics is based on a set of *hishapes* connected by pathways with their corresponding barrier energies. The most straightforward approximation of transition rates can be done using Arrhenius’ equation. Consider the two *hishapesα* and *β*. We initially compute the *hishape* ensemble energy (*ΔG*(*α*),*ΔG*(*β*)) via the *hishapes* partition function contribution calculated by RNAHELICES (see Equation 4). Next, using HIPATH2, we estimate the barrier energy *ΔG*[ *α*,*β*] between the two *hishreps* of *α* and *β*. Finally, we derive the transition rates using Arrhenius’ equation (see equation 5). Using the *hishape* ensemble energy can be seen as weighting the energy by the size of the *hishape* class, which takes into account that the more members a *hishape* has, the higher the probability of a transition into the *hishape*. In contrast, transition out of a large (in terms of members) *hishape* is less likely. Our approach is conceptually similar to the macrostate model introduced with TREEKIN. Here, the folding space is partitioned into macrostates, based on local minima and their basins of attraction. These macrostates are computed by the program BARRIERS, which also computes the transition rates based on the barrier energies. The latter are computed on-the-fly, which is elegant, but has one major drawback: the depth (in terms of free energy above the MFE) of the analysis must be sufficiently large to ensure that saddle points connecting all local minima (macrostates) of interest are present. For real-world examples, this depth can easily reach 10-20 kcal/mol (see Table 2), resulting in a large computational effort to compute the transition rates, especially for long sequences. Our approach circumvents this problem, as the computation of the transition rates is separated from the computation of the macrostates, i.e. *hishapes*, and the latter is more efficient, especially when restricted to strictly negative *hishapes*. Therefore, HIKINETICS is able to simulate folding kinetics for longer sequences than is possible with BARRIERS and TREEKIN. Of course, this ability does not come for free, and we expect our transition rate estimate to be less accurate than the one made using BARRIERS. The results we present in the next section show that this inaccuracy seems to have only a minor influence.

#### Spliced Leader RNA from *Leptomonas collosoma*

The Spliced Leader RNA from *Leptomonas collosoma*[24] has two alternating conformations of nearly equal free energy. Figure 2 shows the results of *hishape* analysis. The two *π*_*m*_*hishapes* ([38] and [27]) represent the two native conformations of the Spliced Leader RNA. The probabilities of conformations 1 and 2 are 0.345271 and 0.470394, respectively, which is in agreement with the bistable character of this RNA.

**Figure 4:**
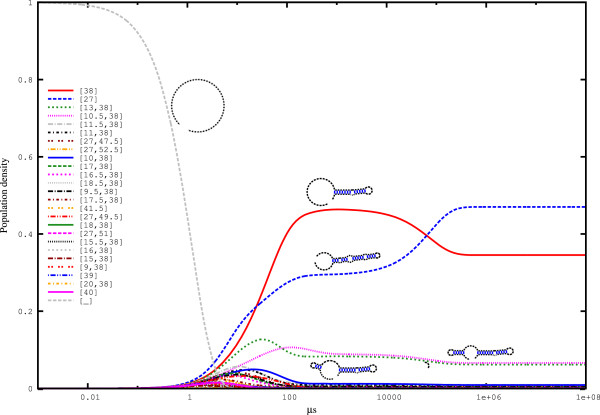
**Folding kinetics of the Spliced Leader RNA from *L. collosoma *simulated with **HIKINETICS**.** Folding kinetics for the 25 best *π*_*h*_*hishapes* plus the open chain ([_]), which was used as the starting structure for this simulation. The simulation was based on the 100 best *π*_*h*_*hishapes* and the open chain. The simulation took 253 min using 64 cores of a 4x AMD Opteron 6282SE machine with 512 GB RAM running under openSuSE 12.2. This figure was generated using. '/HiKinetics.rb -i Input/spliced_leader.seq -o Output/spliced_leader -k 100 -t 4’.

The kinetic analysis in Figure 4 shows that the two major *hishapes* ([38] and [27]) dominate from *t*=10 *μs* until equilibrium. At the end of the simulation, their equilibrium occupancies are the same as the probability calculated by the partition function. Interestingly, both alternative *hishape* classes build plateaus that persist for a long period (from approximately *t*=500 *μs* to *t*=50,000 *μs*) and cross at approximately *t*=50,000 *μs*. If the Spliced Leader RNA degrades within this period, *hishape* [38] would be kinetically preferred, achieving almost 50% occupancy. However, if the lifetime of the Spliced Leader RNA exceeds the time needed to reach equilibrium, *hishape* [27] would win.

**Figure 5:**
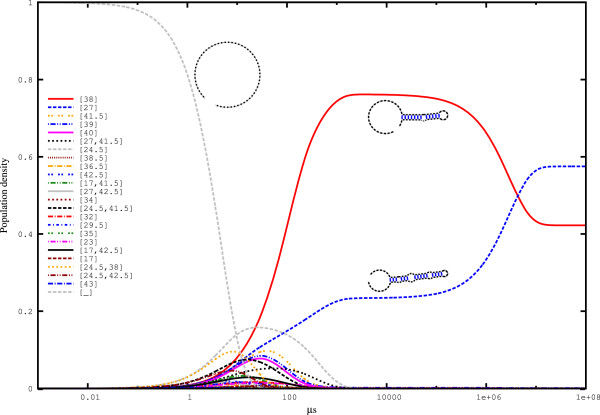
**Folding kinetics of the Spliced Leader RNA from *L. collosoma *simulated with **HIKINETICS** restricted to strictly negative (SN) structures.** The calculation is based on all $\pi_{h}^{\text{SN}}$*hishapes* plus the open chain ([_]), which was used as the starting structure for this simulation. The simulation took 14 min using 64 cores of a 4x AMD Opteron 6282SE machine with 512 GB RAM running under openSuSE 12.2. This figure was generated using. '/HiKinetics.rb -i Input/spliced_leader.seq -o Output/spliced_leader_SN -k 100 -t 4 -s’.

To determine the degree to which strictly negative filtering influences the analysis, we performed a simulation based on strictly negative *hishapes* on the same sequence (see Figure 5). Here, the (arbitrary) timescale of the process is altered, while the characteristics are the same. Note that the two *hishapes* ([13,38] and [10.5,38]), which are related to [38], are not strictly negative and thus are no longer present. As a result of the filtering, the equilibrium probabilities are also altered from 0.345 to 0.422 for *hishape* [38] and from 0.470 to 0.575 for *hishape* [27]. This result is mainly due to the reduced state space, such that each state occurs with higher frequency. Direct computation of the probabilities for the strictly negative *hishapes* using RNAHELICES results in the same values.

**Figure 6:**
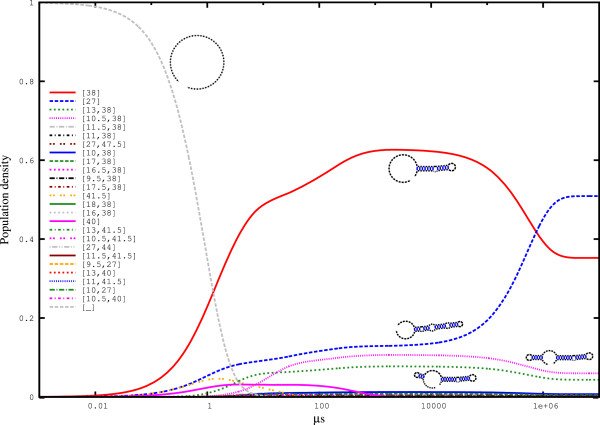
**Folding kinetics of the Spliced Leader RNA from *L. collosoma *simulated with **TREEKIN**.** We applied BARRIERS and TREEKIN to simulate folding kinetics based on the macrostate model. Each macrostate representative local minimum was mapped to its *π*_*h*_*hishape*, and ones with the same *hishape* were merged. The simulation started from the open chain. We show the results for the 25 best *hishapes* plus the open chain.

Next, we compared our *hishape*-based kinetics simulation to the simulation from TREEKIN whose results are shown in Figure 6. Focussing on the two dominant *hishapes* [38] and [27], the similarity to the kinetics based on strictly negative structures (Figure 5) is higher than the similarity to the kinetics for the unrestricted approach (Figure 4). By design, the latter retains more detail, which is reflected by the presence of the two not strictly negative *hishapes* [13,38] and [10.5,38] in this simulation. Again, however, the simulated kinetics is significantly similar to the TREEKIN results. Overall, this result shows that our approach to the simulation of folding kinetics is accurate enough to capture major features of the folding space, such as the late crossing of *hishapes* [38] and [27].

#### The c-di-GMP riboswitch of the *tfoX* from *Candidatus desulforudis audaxviator*

**Figure 7:**
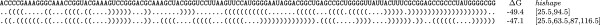
**The alternating structures of the c-di-GMP riboswitch of the *tfoX *gene from *C. desulforudis *audaxviator MP104C.** We took the native structures proposed in [26] and used them as constraints to predict the energetically optimal structure using RNAFOLD. These results were then mapped to the corresponding *hishapes*. *ΔG* is the free energy in *kcal*/*mol*, and *hishape* represents the *π*_*h*_*hishape*.

In the second example, we analysed the c-di-GMP riboswitch of the *tfoX* gene from *Candidatus desulforudis audaxviator* (CP000860.1/c(1860063-1860186), [25]. As shown in Figure 7, it has two states that differ by approixamtely 2.3 kcal/mol in free energy. The c-di-GMP riboswitches, like all riboswitches, are composed of two domains: an aptamer and an expression platform. The aptamer is more conserved and is responsible for binding c-di-GMP, while the expression platform controls expression by alternative conformations. Here, helix 116.5, which is present in the second *hishrep* constitutes a Rho-independent terminator hairpin.

**Figure 8:**
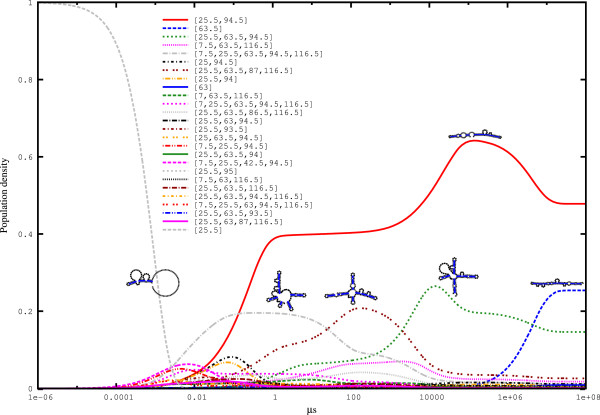
**Simulated folding kinetics of the c-di-GMP riboswitch of the *tfoX *gene from *C. desulforudis *audaxviator MP104C.** The calculation is based on the 100 best $\pi_{h}^{\text{SN}}$*hishapes*, and we used the stable helix ([25.5]) of the aptamer as the initial population. We show the results for the 25 best *hishapes* plus *hishape* [25.5]. The simulation took 24 hours using 64 cores of a 4x AMD Opteron 6282SE machine with 512 GB RAM running under openSuSE 12.2. This figure was generated using. '/HiKinetics.rb -i Input/c_di_GMP_riboswitch.seq -o Output/c_di_GMP_riboswitch_SN -k 100 -t 4 -s -p [25.5]’.

We simulated the folding kinetics based on strictly negative *hishapes* and chose the stable helix ([25.5]) of the aptamer as the initial population (see Figure 8). The *hishape* [25.5,94.5], which corresponds to the native ON conformation, dominates from *t*=0.5 *μs* until thermodynamic equilibrium. Other *hishapes* such as [7.5,25.5,63.5,94.5,116.5], [25.5,63.5,87,116.5], [25.5,63.5,94.5] and [63.5] appear transiently in different periods. The first two correspond to OFF conformations (helix 116.5 is present), and their fraction is significantly increased from approximately *t*=0.01 *μs* to *t*=5,000 *μs*. The *hishape* [25.5,63.5,94.5] likely represents a folding intermediate between the ON and OFF conformations, as it is composed of helices from both structures. Its share increases briefly at time point 10,000 *μs* and drops shortly after, while the fraction of *hishape* [25.5,94.5] increases, which supports the assumption that *hishape* [25.5,63.5,94.5] is a folding intermediate between the ON and OFF conformations. The *hishape* [63.5] appears late (1*e*+06 *μs*) in the simulation. The short time span (*t*=0.01 *μs* to *t*=5,000 *μs*) where OFF conformations achieve a significant fraction of the folding space reflects the kinetic control of this riboswitch [27]. The folding kinetics restricts the time period during which the RNA is accessible for regulation.

## Conclusions

In this paper, we present several methods for improving folding space analysis. First, we introduce strictly negative *hishapes* that represent a reasonable subset of the folding space, i.e., those *hishapes* composed of helices that all have negative energies. We analysed *hishapes* and their strictly negative variant for correspondence to local optima, and found a large overlap. This result supports our idea of using *hishapes* for folding space analysis. Second, we present HIPATH2, an improved algorithm for calculating suboptimal folding pathways between two given secondary structures. A benchmark confirms that HIPATH2 outperforms its predecessor and other heuristics on the chosen dataset. Finally, we present a new approach for simulating RNA kinetics, which is based on *hishapes* and uses HIPATH2 to compute transition rates. The simulated folding kinetics of two well-studied RNAs show that using our approach allows us to draw functional conclusions. The results for the c-di-GMP riboswitch make us wonder if kinetics can help in identifying new riboswitches. To the best of our knowledge, the existing methods for the identification of riboswitches [19,28-31], are based on sequence and/or secondary structure conservation or on structure comparison. No methods use folding kinetics.

Our strategy to disentangle folding space partitioning and barrier energy estimation makes it possible to simulate folding kinetics for fairly long sequences. The most time-consuming step is the computation of pairwise energy barriers using HIPATH2. Because these computations are independent, this step can be easily parallelised, which we already exploited. For massively parallel applications, GPU-accelerated computing is the method of choice, and might be a reasonable option to significantly speed up folding kinetics simulations using HIKINETICS.

## Methods

### Energy parameters

When not mentioned explicitly, we used the most recent set of energy parameters [32].

### Restricting the folding space to strictly negative structures

The algorithm for helix index shape analysis has been developed using Bellman’s GAP [33-35]. Bellman’s GAP supports semantic filtering which filters the answer list with the specified filter function after the objective function is applied. We take advantage of this filtering feature to remove positive energy substructures in the external loop and in multiloops. Because the resulting *hishapes* have negative energy, we term them strictly negative (SN).

### Fuzzy related *hishapes*

The helix index (central position of the loop closing base pair the helix ends in) is susceptible to small variations. If one of the pairing partners shifts by a single position, as in helix slipping, the helix index will also change. Furthermore, in folding pathways between two conformations, intermediate structures may occur that have helices with slightly different helix indices.

To account for these small variations, we introduce a less stringent version of related *hishapes*, which we call fuzzy related *hishapes*.

#### **Definition 1** (**Fuzzy related*hishapes***)

Given two hishapes *α* and *β* in an arbitrary abstraction type and a user-defined threshold *θ*, and letting *ϕ* be a function to extract hairpin loop *helix indices*, fuzzy related *hishapesγ* are the *hishapes* that satisfy

(1) $$\underset{t\in \phi (\gamma )}{\max}\underset{z\in (\phi (\alpha )\cup \phi (\beta ))}{\min}\left|t-z\right|\;\leq \theta$$

### Restricting the number of fuzzy related *hishapes* within HIPATH2

The number of (fuzzy related) *hishapes* has a large impact on the runtime of HIPATH2. For this reason we provide a means to restrict this number. In the previous version (HIPATH), the calculation of related *hishapes* always starts at the most abstract level. If, in this level, the number of *hishapes* is not greater than a user-defined threshold *n*, the next lower abstraction level is used. This step is performed either until the number of *hishapes* is greater than *n* or the user-defined lowest abstraction level *t* is reached. The number of related *hishapes* calculated in this way causes a repeated *hishape* calculation of different abstraction types. For example, if the first attempt does not result in a sufficient number of *hishapes*, they must be calculated for the next abstraction type, and the initial result will be discarded.

To avoid this issue and speed up HIPATH2, we use an auto-adjust strategy that applies the empirically derived formula shown in Equation 2. Precise asymptotics for the number of abstract shapes have been derived in [15,36] and are defined by *a*×*b*^*n*^×*n*^-3/2^ where *n* is the sequence length. We use this formula to adjust the number of related *hishapes* for the HIPATH2 calculations. After empirical testing, we chose *a*×*b*^*n*^=124,000. Therefore, for *n*=500, *k* is approximately 10, which means that we keep the 10 fuzzy related *hishapes* with the lowest free energy. This precaution keeps the HIPATH2 calculation within one hour for two *hishapes* of a 500 nt long sequence.

#### **Definition 2** (**Auto-adjust fuzzy related*hishape* number**).

(2) $$k=124,000\times n^{-3/2}$$

### HIPATH2 algorithm

For the computation of a single pathway between a given start and target structure, we restrict the search space to fuzzy related *hishapes* as defined by Equation 1. Additionally, given an RNA sequence *x*, a start structure *S* and a target structure *T*, only the shortest path from the start to the target structure is computed. Algorithm 1 shows an outline of HIPATH in pseudocode. In line 4, the *N* lowest-energy fuzzy related *hishreps* in the *π*_*h*_ abstraction (-t 1) with respect to the helix index list *H*_*U*_ are calculated using RNAHELICES. In line 7, we use a breadth first search (BFS) to estimate the energy barrier between *L*[ *i*] and *L*[ *j*], which is stored in the matrix *M*_*BFS*_ at position (*i*,*j*). In line 10, we apply a modified version of Dijkstra’s algorithm [37] in which the edges are weighted with the barrier energies calculated by the BFS algorithm. Instead of computing the sum of the weights, we take the maximum weight along the path and look for the path with the lowest maximum weight.

#### Algorithm 1 **HiPath2 (rna*s*,structure*S*,structure*T*)**

### Kinetic folding simulation

In the following section, we describe how the folding kinetics of an RNA molecule is calculated from the barrier energies between *hishapes*. In [4], the authors introduced a partitioning of the conformation space based on gradient basins of the local energy minima. The authors term these partitions *macrostates* and use the macrostate ensemble free energy to compute the transition rates. In our simulation, we divide the conformation space into *hishapes*. For each *hishapeα*∈, we compute the ensemble free energy based on the partition function [38], where *ΔG*(*x*) represents the free energy of conformation *x*, *k* is the universal gas constant, and *T* is the absolute temperature in Kelvin.

(3) $$Z_{\alpha }=\underset{x\in \alpha }{\sum }e^{-\mathrm{\Delta G}(x)/\text{kT}}$$

and the corresponding *hishape* ensemble energy

(4) $$\Delta G_{\alpha }=-\text{kTln}Z_{\alpha }$$

Between the two *hishapesα* and *β*, we approximate the transition rates using Arrhenius’ equation,

(5) $$r_{\beta \alpha }=Ae^{-(\mathrm{\Delta G}[\alpha ,\beta ]-\mathrm{\Delta G}(\alpha ))/\text{kT}}$$

where *ΔG*[ *α*,*β*] is the barrier energy between the two *hishreps* of *α* and *β* computed by HIPATH2. The pre-exponential factor *A* can be determined by fitting the available experimental data to the formula $\log k_{F}=Ae^{-aN^{b}}$, where *k*_*F*_ is the experimentally determined folding rate, and *N* is the number of nucleotides. In [39], a value of *A*=1.0 *μ*s^-1^ was proposed, which we use for all our simulations.

Let *p*_*α*_(*t*) be the probability of a conformation to be in *hishapeα* at time *t*, and the probability distribution can be computed by the master equation

(6) $$\frac{dp_{\alpha }(t)}{\text{dt}}=\underset{\beta \in ℋ}{\sum }p_{\beta }(t)r_{\alpha \beta },\text{with}\;\;r_{\alpha \alpha }=-\underset{\beta \neq \alpha }{\sum }r_{\beta \alpha }$$

The equation can be rewritten in matrix form

(7) $$\frac{d}{\text{dt}}\vec{p}(t)=R\vec{p}(t)$$

From the matrix differential equation, the folding kinetics are described by (8) where $\vec{p}(0)$ is the initial distribution of the CTMC.

(8) $$\vec{p}(t)=\vec{p}(0)e^{\text{tR}}$$

## Competing interests

The authors declare that they have no competing interests.

## Authors’ contributions

JH implemented the software, performed all the computations and drafted the manuscript. BV designed the study and wrote the manuscript. Both authors have read and approved the final manuscript.
